# Step Enzymatic Hydrolysis and *In Silico* Screening-Assisted Preparation of Bioactive Peptides from Abalone

**DOI:** 10.3390/foods14071209

**Published:** 2025-03-29

**Authors:** Kanzhen Liu, Cuiping Pang, Qinghua Li, Jianghua Li, Guocheng Du, Guoqiang Zhang

**Affiliations:** 1 Science Center for Future Foods, Jiangnan University, 1800 Lihu Road, Wuxi 214122, China; 6220201082@stu.jiangnan.edu.cn (K.L.); 8202407012@jiangnan.edu.cn (Q.L.); lijianghua@jiangnan.edu.cn (J.L.); gcdu@jiangnan.edu.cn (G.D.); 2 School of Biotechnology, Jiangnan University, 1800 Lihu Road, Wuxi 214122, China; 3 Shenzhen Institute of Synthetic Biology, Shenzhen Institute of Advanced Technology, Chinese Academy of Sciences, Shenzhen 518055, China; cp.pang@siat.ac.cn; 4 Key Laboratory of Industrial Biotechnology of Ministry of Education, Jiangnan University, 1800 Lihu Road, Wuxi 214122, China; 5 Jiangsu Province Engineering Research Center of Food Synthetic Biotechnology, Jiangnan University, 1800 Lihu Road, Wuxi 214122, China

**Keywords:** abalone, enzymatic hydrolysis, bioactive peptides, virtual screening, molecular docking

## Abstract

Bioactive components of abalone and other marine organisms have attracted significant attention owing to their functional performance. The development of peptides with bioactivity like angiotensin-converting enzyme inhibitory (ACEi) and antioxidant properties is of great significance for chronic disease management and drug discovery. In this study, according to the issues of low utilization rate and bioactive content from the hydrolysate of abalone, single-factor and orthogonal experiments were designed to improve the utilization rate of abalone protein, and step hydrolysis with specific proteases was used to improve the overall biological activity of the hydrolysate. A total of 1937 peptide sequences were obtained from the highly bioactive components after separation and peptidomic analysis. Through virtual screening and molecular docking, 14 peptides exhibiting ACEi activity were identified and synthesized for experimental verification, with IC_50_ values ranging from 0.05 to 0.54 mg/mL. Notably, nine of these peptides were powerful antioxidants. The developed step enzymatic hydrolysis and *in silico* screening-assisted preparation also provided a feasible and efficient method for exploring more bioactive peptides from diverse biomasses.

## 1. Introduction

The marine environment is a relatively undeveloped source of functional ingredients and marine-derived chemicals as functional food ingredients, which play an important role in maintaining health and preventing chronic diseases [1]. With the public’s pursuit of health, bioactive peptides from marine sources have become a popular research focus in food, medicine, and industry due to their diverse functions, wide availability, strong specificity, and low toxicity [2]. For example, the high-quality and efficient angiotensin-converting enzyme inhibitory (ACEi) peptides were obtained from *Mytilus edulis* [3], *Antarctic krill* [4], and other marine organisms. Antioxidant peptides can also be obtained from a wide range of marine sources, such as algae, mollusks, and fish [5]. They can reduce the incidence of cardiovascular disease and hypertension by regulating reactive oxygen species production, enzyme activity, and antioxidant pathways [6].

As a high-nutritional-value single-shell mollusk, the abalone is high in energy and extremely rich in concentrated marine proteins, vitamins, and trace elements. It is a high-quality protein source for supplementing essential amino acids such as lysine and threonine [7]. Many studies have also shown that abalone is rich in peptides, polysaccharides, free amino acids, fatty acids, and other active ingredients [8], and these active substances have anticoagulation, anti-tumor, anti-inflammatory, and hypoglycemic effects [9]. Abalone viscera are byproducts of food processing and are usually discarded as waste. Studies on the active ingredients of abalones have mostly focused on processing visceral waste to improve its added value. The functional polysaccharides which showed excellent antioxidant activity [10] and immunoregulation ability [11] were obtained from abalone viscera. The lipid components [12], such as phospholipids [13], in abalone viscera have biological activities similar to those of abalone visceral polysaccharides. In addition, the abalone foot muscle proteins were hydrolyzed to analyze the solubility, emulsification stability index, foaming stability, and other indicators of the hydrolysates [14]. However, studies on the comprehensive utilization of abalones for bioactive peptide preparation are lacking.

Improving the yield and bioactivity of peptides has always been a research hotspot, where an efficient and controllable enzyme hydrolysis process is crucial for polypeptide production [15]. To efficiently hydrolyze seaweed protein and produce ACEi peptides, a glycolytic enzyme was first used to degrade polysaccharides before protease hydrolysis, which increased the protein content by 2.91-fold [16]. To obtain specific bioactive peptides with high purity and activity, ultrafiltration, gel permeation chromatography (GPC), and high-performance liquid chromatography (HPLC) have been used for separation and purification, and peptide sequences have been identified using mass spectrometry [17]. Six novel ACEi peptides were identified by stepwise separation of protein hydrolysate from skipjack tuna muscle and revealed the active mechanism using molecular docking [18]. Recently, the development of peptidomics and bioinformatics techniques has significantly accelerated the discovery and analysis of food-derived bioactive peptides [19].

In this study, abalone meat and viscera were used as the raw materials for primary enzymatic hydrolysis, in which enzymes conducive to improving protein utilization were used. According to the amino acid sites speculated to be related to biological activity based on the separation and analysis of primary enzymatic hydrolysis product, three specific proteases which have specific hydrolysis sites were selected for the second step of enzymatic hydrolysis to improve biological activity. A combination of functional analysis, peptidomics, and molecular docking was used to fully explore and evaluate the functional peptides.

## 2. Materials and Methods

### 2.1. Materials

Haliotis discus hannai was obtained from Fujian, China; lipase (>30,000 μ/g) from Yuanye Biotechnology Co., Ltd., Shanghai, China; and papain (>200,000 μ/g) from Xiameng Biotechnology Co., Ltd., Shenzhen, China. Neutral protease (>2.4 au-a/g) and alkaline protease (>6.5 au-n/g) were purchased from Novozyme Biotechnology Co., Ltd. (China) and trypsin (>250,000 μ/g) from Shanghai McLean Biochemical Technology Co., Ltd., Tianjin, China. Prolyl endopeptidase (>25 μ/mL) was obtained from Xiasheng Enzyme Biotechnology Co., Ltd., Cangzhou, China. α-Chymotrypsin (>35,000 μ/g) was sourced from bovine pancreas, and proteinase K (>500 μ/mL) was derived from Candida albicans Linnaeus (Aladdin Biochemical Technology Co., Ltd., Shanghai, China).

### 2.2. Preparation of Abalone Dry Powder and Abalone Enzymatic Hydrolysates

The abalone meat was boiled with water at 110 °C for 4 h, then cooled and frozen, lyophilized to constant weight in a freeze dryer (Xinzhi Biotechnology Co., Ltd., Ningbo, China), ground, and filtered through a 0.22 mm mesh screen, yielding dry abalone powder. The total protein content of the powder was determined using the Kjeldahl method. The dried abalone powder was mixed with ultrapure water and enzymatically hydrolyzed by various proteases under different conditions for 6 h. To maintain the pH between 6.5 and 7.5, 0.1 mol/L NaOH or 0.1 mol/L HCl was added every 30 min. The enzymatic hydrolysate was collected, heated at 90 °C for 10 min to inactivate the enzyme, and centrifuged at 12,000 rpm for 10 min to separate the supernatant. A clearer supernatant indicates better peptide separation. After freeze-drying, the supernatant was made into powder.

The degree of hydrolysis (DH) was determined using the o-phthalaldehyde (OPA) method. OPA regent and serine standard solution were prepared according to previous research [20]. The testing method are as follows:

Separately mixed 400 μL of the serine standard solution, ultrapure water, and the sample solution with 3 mL OPA reagent. The mixed solutions were precisely reacted in the dark for 2 min. The absorption values at 340 nm wavelength were ODstand, ODblank and ODsample, respectively. DH (%) was calculated using Equations (1) and (2):

(1) $$\mathrm{Serine}\;{\mathrm{NH}}_{2}=\frac{\mathrm{ODsample}-\mathrm{ODblank}}{\mathrm{ODstand}-\mathrm{ODblank}}\times \;0.9516\;\mathrm{meqv}\cdot L^{-1}\;\times \;\frac{0.1\;\times \;100}{X\;\times \;P}\;L\cdot g^{-1}\;\mathrm{protein}$$

In Equation (1): Serine NH_2_ was meqv serine NH_2_·g^−1^ protein; 0.1 was 0.1 L (sample volume); 100 was dilution ratio; *X* was sample quality; and *P* was protein content (%).

(2) $$\mathrm{DH}/\%=\frac{\mathrm{Serine}\;{\mathrm{NH}}_{2}-\beta }{\alpha \;\times \;h_{\mathrm{tot}}}\;\times \;100\%$$

In Equation (2): α was 1.00; β was 0.40; and h_tot_ was 8.6 [20].

### 2.3. Optimization of Enzymatic Hydrolysis of Abalone

Enzymatic hydrolysis was performed using 0.1% lipase, 0.1% papain, 0.2% neutral protease, 0.2% trypsin, 0.2% alkaline protease, or 0.2% neutral protease + 0.2% trypsin + 0.2% alkaline protease. The solid–liquid ratio of abalone was 1:20, the temperature was 55 °C, and the enzymatic hydrolysis time was 6 h. Samples were removed at 2, 4, and 6 h to determine DH (the same as Section 2.2).

The enzyme ratio was fixed, and the enzymatic hydrolysis conditions were optimized using a single-factor experiment. Hydrolysis times of 2, 3, 4, 5, and 6 h; enzyme dosages of 0.5%, 1%, 2%, 3%, and 4%; hydrolysis temperatures of 30, 37, 46, 55, and 60 °C; and solid–liquid ratios of 1:5, 1:10, 1:20, 1:30, and 1:40 were chosen for this study, and DH was measured by sampling (as described in Section 2.2).

According to the results of the single-factor experiment, hydrolysis time, hydrolysis temperature, solid–liquid ratio, and enzyme dosage were chosen for investigation in the orthogonal experiment. The DH and polypeptide content of polypeptides (200–10,000 Da) in the enzymatic hydrolysate supernatant were comprehensively evaluated. An orthogonal experimental table with four factors and three levels was used. The arrangement of the factor levels is presented in Table 1. Polypeptide content was determined using HPLC protein molecular weight (MW) distribution analysis [21]. The results of the orthogonal test were presented in Table 2. Three validation experiments were performed according to the optimal enzymatic hydrolysis conditions (Table 3).

The initial product of abalone hydrolysis (AHIP) was hydrolyzed by adding specific proteases under the conditions of a single-, double-, and triple-enzyme combination at 1% of the total enzyme dosage and other hydrolysis conditions, referring to the optimal enzymatic hydrolysis conditions. The ACEi and ABTS antioxidant activities of the hydrolysates were determined at 1 mg/mL.

### 2.4. HPLC Peptide MW Distribution

HPLC was used to determine the MW distributions of the hydrolysis products. Cytochrome C (12,384 Da), aprotinin (6500 Da), bacteriocin (1422 Da), arginine–tyrosine–arginine (451 Da), and arginine–arginine (189 Da) were used as standards. The chromatographic column was a TSK gel 2000 SWXL (300 mm × 7.8 mm); the mobile phase was composed of acetonitrile/water/trifluoroacetic acid, 36/64/0.1 (*V*/*V*); the column temperature was 30 °C; the injection volume was 10 μL; the detection wavelength was 205 nm; the flow rate was 1 mL/min; and the detection time was 15 min. For sample processing, the freeze-dried powder was thawed and dissolved to prepare a 1% (*w*/*v*) solution. After centrifugation, the sample was passed through a 0.45 μm water film for detection.

### 2.5. Separation of Enzymatic Hydrolysis Products and Determination of Amino Acid Composition

After the optimal enzymatic hydrolysis process, the obtained enzymatic supernatant was freeze-dried to constant weight and stored at 4 °C for future use. The crude abalone peptide product was centrifugated by 4000 rpm at 25 °C using MWCO ultrafiltration membranes [18] of 3 kDa and 10 kDa and divided into three components of >10 kDa, 3~10 kDa, and 0~3 kDa. The products were freeze dried to constant weight and stored at 4 °C after their biological activities were determined.

The components (5 mL, 50 mg/mL) obtained by ultrafiltration were separated on Sephadex G-25 and Sephadex G-15 columns (2.6 × 40 cm) eluted with ultrapure water at a flow rate of 0.6 mL/min. The absorbance of the eluent was monitored at 280 nm. The fractions under the different peaks were collected, freeze dried to constant weight, and stored at –20 °C after their biological activities were determined.

The amino acid composition of the GPC components was determined according to a previously published method [22]. The sample loading quantity was 100.0 mg (solid)/1.0 mL (liquid); mobile phase A (pH 7.2): 27.6 mmol·L^−1^ sodium acetate–triethylamine–tetrahydrofuran (500/0.11/2.5, *v*·*v*^−1^); mobile phase B (pH 7.2): 80.9 mmol·L^−1^ sodium acetate–methanol–acetonitrile (1/2/2, *v*·*v*^−1^); Agilent Hypersil ODS column (5 μm, 4.0 mm × 250 mm); gradient elution was adopted (elution program: 0 min, 8% B; 17 min, 50% B; 20.1 min, 100% B; 24.0 min, 0% B); flow rate was 1.0 mL·min^−1^; column temperature 40 °C; UV detection wavelength was 338 nm, proline was detected at 262 nm; amino acid content was quantified by the external standard method.

### 2.6. Determination of ACE Inhibitory Activity and ABTS Antioxidant Activity

ACEi activity was determined according to the method described by Ye et al. [23], with some modifications. Using FAPGG as the substrate, 10 μL ACE (0.1 μ/mL), 50 μL FAPGG (1 mmol/L, dissolved in 0.1 M borate buffer, pH 8.3), and 40 μL of sample were added to the enzyme plate. Using a microplate reader at 340 nm, the absorbance A_1_ before the reaction was measured, and then the absorbance A_2_ was measured after incubation at 37 °C in the dark for 30 min. ΔA = A_1_ − A_2_ was calculated. ACE activity was expressed as the change in absorbance per unit time. Borate buffer was used as a control. The ACE inhibition rate (%) was calculated using Equation (3):

ACE inhibitory rate (%) = (1 − ΔA_sample_/ΔA_blank_) × 100% (3)

ABTS antioxidant activity was determined according to the method described by Wang et al. [24], with some modifications. ABTS^+^ working solution was prepared by mixing ABTS solution (7.0 mM) with an equal volume of potassium persulfate solution (2.45 mM), incubating at room temperature in the dark for 12–16 h, and diluted by 50% ethanol solution to 0.75~0.80 absorbance at 734 nm. Then, 200 μL ABTS^+^ working solution was added to 10 μL of sample, and the solution was incubated at room temperature in the dark for 30 min. The absorbance at 734 nm was measured. PBS was used as the control. The ABTS^+^ clearance rate (%) was calculated using Equation (4):

ABTS^+^ clearance rate (%) = (A_blank_ − A_sample_)/A_blank_ × 100% (4)

These two methods can be used to calculate the IC_50_ and EC_50_ values of 50% biological activity through linear regression analysis of the activity concentration curve.

### 2.7. Peptidomic Analysis

Label-free quantification is a direct MS analysis of protein–enzymatic peptides using liquid chromatography–mass spectrometry. By analyzing the MS data generated in the large-scale identification of proteins, we compared the signal intensity of the corresponding peptide in different samples to quantify the protein corresponding to the peptide. Sample processing included peptide precipitation, filtration, decontamination, desalting, mass, and data dependent acquisition qualitative database construction. The analysis process included raw data quality control as well as qualitative and quantitative analyses of the peptides. This process was completed by China Guangzhou Gene Denovo Biotechnology Co., Ltd., Guangzhou, China.

### 2.8. Molecular Docking

Molecular docking was performed using AutoDock Vina 1.1.2 software [25]. The crystal structures of the human ACE–lisinopril complex (1O86) and Keap1 (2FLU) were obtained from the RCSB Protein Data Bank (https://www.rcsb.org/, accessed on 1 October 2024). Polypeptide molecules were constructed using PyMOL 2.6 and subjected to energy minimization. PyMOL 2.6 was used to treat proteins, remove water molecules and small-molecule ligands, and add hydrogen. Receptor proteins and peptides were converted to PDBQT format using AutoDock Tools. The docking box center was defined according to the crystal ligand position, and the box side length was 40 Å. The highest Vina docking score was chosen as the final result, and visual analysis was performed using PyMOL 2.6.

### 2.9. PeptideRanker Tool for Screening Potential Bioactive Peptides

Peptide sequences were entered into the PeptideRanker website (http://distilldeep.ucd.ie/PeptideRanker/, accessed on 1 September 2024) to predict the probability of polypeptide activity [26]. Peptide synthesis was completed by Modif Biotechnology Co., Ltd., Suzhou, China.

### 2.10. Statistical Analysis

All experiments were independently repeated more than three times, and the results were expressed as the mean ± standard deviation. The software spss19.0 was used for one-way analysis of variance (*p* < 0.05).

## 3. Results

### 3.1. Establishment and Optimization of Enzymatic Hydrolysis Process

Enzymatic hydrolysis is the most important step in preparing bioactive peptides [27]. In contrast to other studies that decreased the raw materials and extracted proteins, such as the study conducted by Sitanggang et al. [28], whole abalone after shelling, boiling, and freeze drying was used as the substrate in this study, reducing the complex protein-extraction steps. In the enzymatic hydrolysis process, lipase can break the triglyceride bond and release proteins from meat, making the peptide bond more exposed and susceptible to proteases [29]. Papain softens meat [30], which is conducive to the combination of enzymes and abalone. Neutral protease [31], trypsin [32], and alkaline protease [18] exhibit excellent performance in the preparation of ACEi and antioxidant peptides.

**Figure 1:**
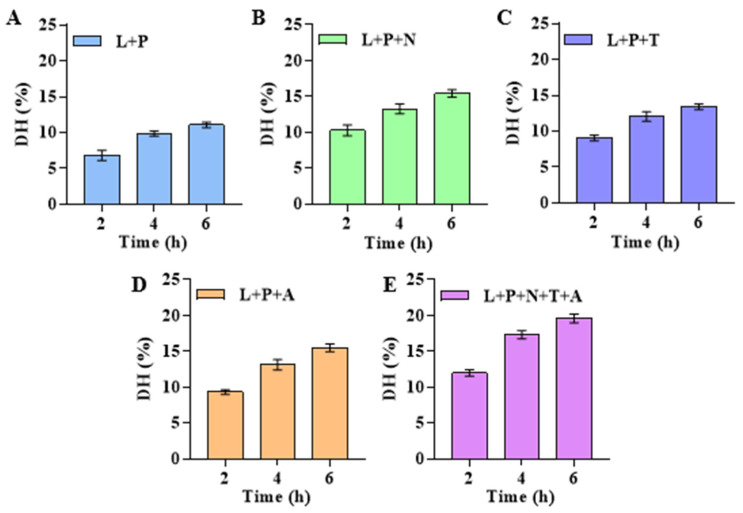
Effect of different enzyme combinations on DH of abalone. (**A**) L + P: 0.1% lipase and 0.1% papain. (**B**) L + P + N: 0.1% lipase, 0.1% papain, and 0.2% neutral protease. (**C**) L + P + T: 0.1% lipase, 0.1% papain, and 0.2% trypsin. (**D**) L + P + A: 0.1% lipase, 0.1% papain, and 0.2% alkaline protease. (**E**) L + P + N + T + A: 0.1% lipase, 0.1% papain, 0.2% neutral protease, 0.2% neutral protease, 0.2% trypsin, and 0.2% alkaline protease.

Firstly, the optimal enzyme ratio was determined, and then single-factor experiments on enzymatic hydrolysis time, enzyme dosage, temperature, and solid–liquid ratio were completed. Based on this, an orthogonal experiment of three factors and four levels with multiple evaluation indices was designed to determine the optimal enzymatic hydrolysis process. Compared with the other combinations (Figure 1A–D), when lipase, papain, neutral protease, trypsin, and alkaline protease were combined to hydrolyze dry abalone powder (Figure 1E), the DH of abalone protein was the highest (19.5% at 4 h). Therefore, the lipase: papain: neutral protease: alkaline protease: trypsin ratio was set to 1:1:2:2:2.

Secondly, the single-factor experiment showed that the DH increased with higher curve slope in the optimal ranges of hydrolysis time (3–5 h), enzyme dosage (1–3%), hydrolysis temperature (37–55 °C) and solid–liquid ratio (1:10–30) (Figure S1A–D). The DH was positively correlated with enzymatic hydrolysis time and enzyme dosage, but polypeptide content tended to initially increase then decrease; this trend was probably due to the increased product concentration caused by excessive hydrolysis, which would inhibit protein hydrolysis [33]. In addition, the changing trend of the biological activity of the product was similar to that of the polypeptide content because excessive hydrolysis would cause the generated active substances to decompose and lose activity [34].

According to the single-factor results, the factors and levels of the orthogonal design were determined (Table 1). The DH of abalone protein and polypeptide content of the enzymatic hydrolysis products were used as evaluation indices, where a high DH and high polypeptide content (so as to avoid excessive protein hydrolysis) were maintained. To ensure the unity of each of these indices, the best index of the experimental results was set at 50 and 50 points, respectively, and the comprehensive score was 100 points. As shown in Table 2, hydrolysis temperature and enzyme dosage had the greatest influence on the abalone protein DH and polypeptide content in the enzymatic hydrolysate, followed by hydrolysis time and solid–liquid ratio, with hydrolysis time having the least influence. As listed in Table 2, the optimal enzymatic hydrolysis conditions according to the orthogonal experiment are a hydrolysis time of 4 h, hydrolysis temperature of 55 °C, solid–liquid ratio of 1:30, and enzyme dosage of 3% (*g*/*g*).

To determine the reliability and stability of the abalone enzymatic hydrolysis process, validation experiments were performed. The results showed DH values of 21.1%, 21.6%, and 23.5% and polypeptide contents of 70.7%, 73.4%, and 73.2%. The RSD of each evaluation index in the validation experiments was less than 3%. In other studies, the enzymatic hydrolysis process of tilapia leftovers was optimized by response surface methodology, with only the DH as the index, and the DH reached 36.2% [35]. Although the DH in orthogonal experiment 7 (Table 2) reached 29.6%, the polypeptide content was the lowest, at 63.4%. Improving the DH of proteins and ensuring a high yield of marine bioactive peptides is in line with the development concepts of marine biotechnology and pharmacology [36].

### 3.2. Separation and Analysis of Bioactive Abalone Peptides

**Figure 2:**
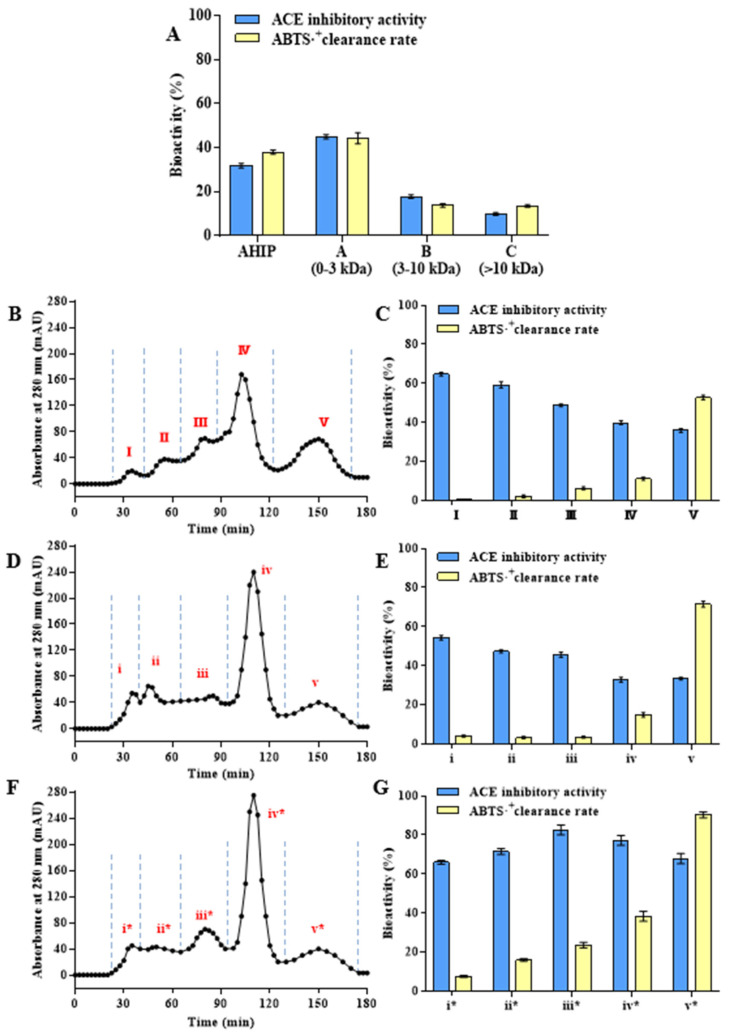
Separation of enzymatic hydrolysis products and determination of biological activity of 1 mg/mL subfractions. (**A**) Bioactivity of AHIP and its ultrafiltration subfractions. (**B**) Chromatogram of AHIP-A separated by Sephadex G-25. (**C**) Bioactivity of subfractions I, II, III, IV, and V separated from AHIP-A by Sephadex G-25. (**D**) Chromatogram of AHIP-A separated by Sephadex G-15. (**E**) Bioactivity of subfractions i, ii, iii, iv, and v separated from AHIP-A by Sephadex G-15. (**F**) Chromatogram of the secondary hydrolysate of abalone peptide (referred to as APSH) separated by Sephadex G-15. (**G**) Bioactivity of subfractions i*, ii*, iii*, iv*, and v* separated from APSH by Sephadex G-15.

The dried abalone powder was treated using the optimal enzymatic hydrolysis process, and the supernatant of the obtained enzymatic hydrolysis solution was centrifuged and freeze dried to obtain AHIP. The ACEi and ABTS antioxidant activities of 1 mg/mL AHIP were 31.6% and 38.13%, respectively (Figure 2A). Compared with previously reported studies, the AHIP prepared in this study did not have high ACEi or antioxidant activity; however, it demonstrated higher protein utilization and peptide yield. The polypeptide solution prepared via enzymatic hydrolysis is not always highly active because the composition of the solution is complex, and most peptides have similarities [37]. To explore the differences in peptide components in different MW ranges, the crude products of the abalone peptides were separated using ultrafiltration and GPC, and their amino acid compositions were analyzed.

First, AHIP was separated into three components: AHIP-A (0–3 kDa), AHIP-B (3–10 kDa), and AHIP-C (>10 kDa) by ultrafiltration. As shown in Figure 2A, the ACEi activity (45.13%) and ABTS antioxidant activity (44.05%) of 1 mg/mL AHIP-A dry powder were higher than those of AHIP, AHIP-B, and AHIP-C. Almost all bioactive peptides obtained from marine organisms by enzymatic hydrolysis have MWs below 3 kDa [5]. To further enrich the highly bioactive components, Sephadex G-25 (1–5 kDa) and Sephadex G-15 (0–1.5 kDa) GPC (Figure 2B,D) were used to further separate the AHIP-A. In this way, the obtained components contained peptides with similar molecular weights and chain lengths, which could be used for further analysis. AHIP-A was separated into A-I–V and A-i–v according to MW, and the biological activities of all components were determined at 1 mg/mL. Interestingly, in the 0–3 kDa range, the high-molecular-weight components A-I (64.5%) and A-i (54.3%) had the highest ACEi activities, while the low-molecular-weight components A-V (52.3%) and A-v (71.5%) had the highest ABTS antioxidant activities (Figure 2C,E). In previous studies, medium-long peptides with a MW range of 2–3 kDa (A-I and A-i) were cut off to produce lower-MW peptides with high activities [38]. Given the advantages of gastrointestinal tract absorption and high bioactivity, most researchers have focused on peptides smaller than 1 kDa [39]. During *in vitro* bioactivity evaluation, smaller peptides exhibit superior penetration capacity into the active pockets/binding sites of target substrates, consequently exerting their bioactivity. However, medium-long peptides tend to have low but relatively stable bioactivity [18], which is of research significance.

The biological activity of polypeptides was closely related to their amino acid composition [40]. Therefore, the amino acid composition of highly bioactive ingredients A-I (2–3 kDa) and A-V (0–1 kDa) were analyzed (Table 4). The results showed that the high-ACEi bioactive components contained more glycine (Gly), alanine (Ala), methionine (Met), lysine (Lys), proline (Pro), and tryptophan (Trp), while the high ABTS antioxidant bioactive components contained more tyrosine (Tyr), cysteine (Cys), and phenylalanine (Phe). The higher contents of hydrophobic amino acids, aromatic amino acids, and proline are consistent with the ACEi peptide model [41]. Based on the difference in amino acid composition between A-I and A-V, it was presumed that more ACEi peptides were not fully digested or released.

### 3.3. Improvement of Bioactivity of Abalone Polypeptides by Secondary Enzymatic Hydrolysis with Specific Proteases

**Figure 3:**
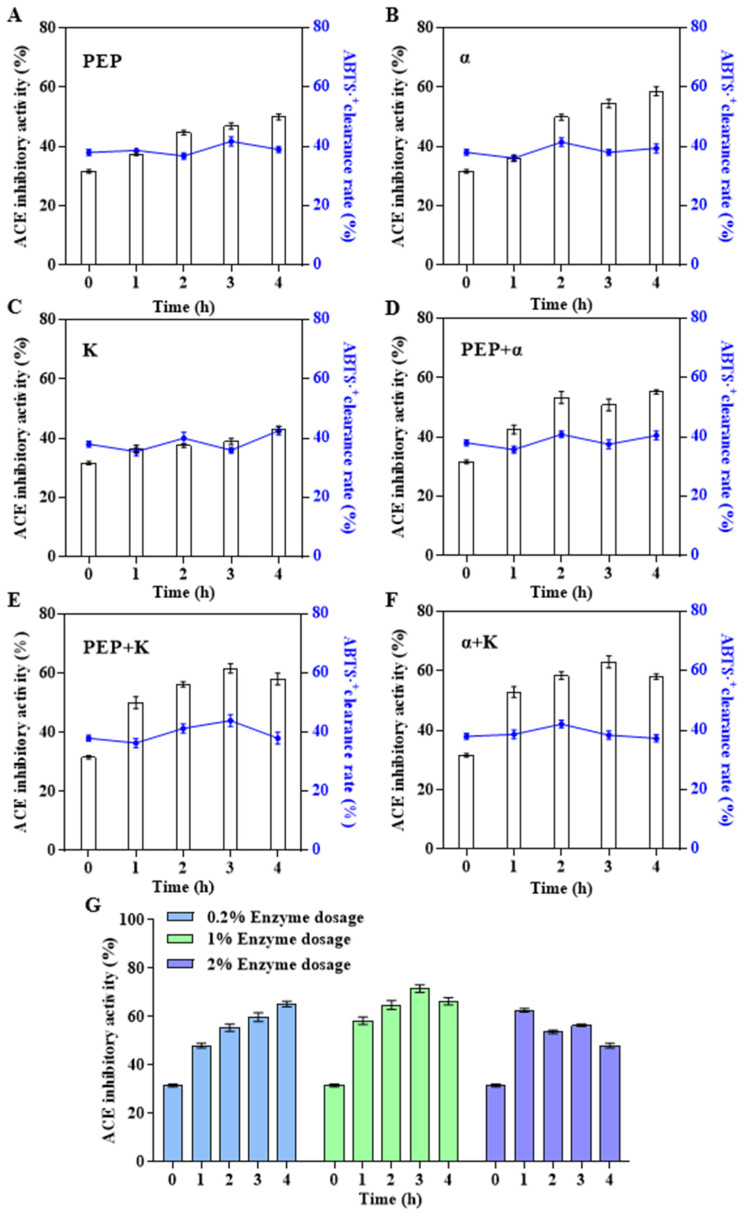
Effect of different specific protease combinations on biological activity of AHIP. (**A**) PEP: 1% prolyl endopeptidase. (**B**) α: 1% α-chymotrypsin. (**C**) K: 1% proteinase K. (**D**) PEP + α: 0.5% prolyl endopeptidase and 0.5% α-chymotrypsin. (**E**) PEP + K: 0.5% prolyl endopeptidase and 0.5% proteinase K. (**F**) α + K: 0.5% α-chymotrypsin and 0.5% proteinase K. (**G**) Prolyl endopeptidase: α-chymotrypsin: proteinase K = 1:2:1. The bioactivity of 1 mg/mL hydrolysate is shown in all figures.

To promote the release of functional peptides, three relatively specific proteases—prolyl endopeptidase (PEP), α-chymotrypsin, and proteinase K—were used to improve the ACEi activity of abalone polypeptides. PEP is a protease that specifically hydrolyses peptide bonds on the carboxyl side of proline [42]. However, PEP has rarely been used to prepare bioactive marine peptides. The important role of tryptophan in ACEi peptide was verified by *in vitro* enzymatic hydrolysis experiments, using α-chymotrypsin, which has strong specificity for tryptophan, and proteinase K, which has strong specificity for hydrophobic amino acids and aromatic amino acids, to combine enzymatic hydrolysis; this approach significantly improved the ACEi activity of the product [43]. First, a single specific protease (Figure 3A–C) or two specific proteases (Figure 3D–F) were used to hydrolyze AHIP. The results showed that the effect of α-chymotrypsin on improving the ACEi activity of abalone polypeptides was higher than that of PEP and proteinase K. ACEi activity was enhanced by combining the two enzymes and was higher than that of a single enzyme. When the total amount of enzymes was 1% (*v*/*v*), the α-chymotrypsin: PEP: proteinase K ratio was 2:1:1, and the hydrolysis time was 3 h; the ACEi activity of the secondary hydrolysate abalone peptide (referred to as APSH) was maximized to 72.14%, which was ≈2.3-fold higher than that of the abalone polypeptide before enzymatic hydrolysis (Figure 3G). When the three specific proteases directly hydrolyzed abalone powder or combined with the complex enzymes in the first step (Figure S2), the ACEi activities of the hydrolysates were similar or even lower. It was speculated that the specific proteases might have higher requirements for the substrate and be suitable for secondary hydrolysates. Stepwise enzymatic hydrolysis was found to be more advantageous for the preparation of smaller active fragments [44]. In addition, in the three enzyme hydrolysis experiments, the ACEi activity of the abalone polypeptide first increased and then decreased owing to excessive hydrolysis, indirectly proving the importance of the aforementioned amino acids in the ACEi polypeptide sequence.

Using the same method, APSH was successively separated using ultrafiltration (0–3 kDa) and GPC (Sephadex G-15) to obtain the peptide components with higher biological activities (Figure 2F). Compared with AHIP, the GPC components obtained in the 0–3 kDa range of APSH gave rise to similar peaks, and the peak areas of the relatively high-MW components (i, ii → i*, ii*) were significantly reduced, while the peak areas of the lower-MW components (iii, iv → iii*, iv*) were significantly increased. In particular, the ACEi activity of the components APSH-i*–v* was significantly increased and that of APSH-iii* was 83.03% (Figure 2G). Taken together, these results fully prove the feasibility of the second-step enzymatic hydrolysis of AHIP with specific proteases to improve the overall biological activity of the peptide components.

### 3.4. Peptidomic Analysis of APSH-iii* and APSH-iv*

**Figure 4:**
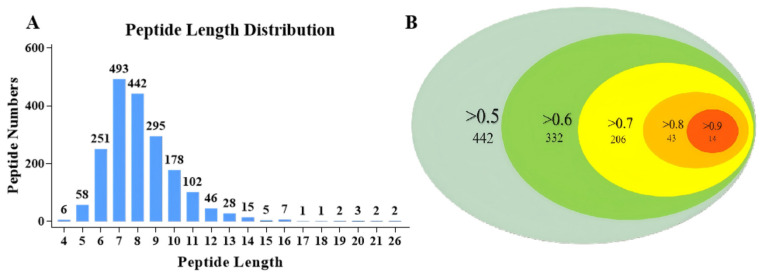
Peptidomics analysis and PeptideRanker prediction. (**A**) Length distribution of peptides contained in APSH-iii* and APSH-iv*. (**B**) Schematic of PeptideRanker prediction results, including the probability of biological activity and the number of corresponding peptides.

Using peptidomics analysis based on LC–MS/MS, all the peptide sequences in APSH-iii* (highest ACEi activity) and APSH-iv* (highest peptide abundance) were quantitatively analyzed. According to the peptide identification results, 1937 polypeptides were detected in the two components, of which 90% were 0–10 amino acids in length (Figure 4A). The sophisticated peptide-separation technologies such as reversed-phase HPLC were used to separate the highly bioactive components obtained from GPC [18]. However, only a small number of peptides can be separated, and not all peptides exhibit biological activities. All peptides contained in the complex polypeptide components were mined using peptidomics, and the results were used as the initial database to screen for bioactive peptides [45]. This approach not only enabled comprehensive and deep peptide mining but also avoided experimental contingency.

### 3.5. Virtual Screening of Bioactive Peptides Using Bioinformatics Tools and Molecular Docking

Bioinformatics tools and molecular docking technology were used to virtually screen ACEi peptides in the database, which contained 1937 polypeptides obtained from peptidomics analysis. The obtained peptide sequences were predicted for biological activity in PeptideRanker (Table 5). Since PeptideRanker regards peptides with scores above 0.5 as bioactive by default [46], this study summarizes the peptide sequences of different score intervals to clearly visualize the distribution of potential bioactive peptides. As shown in Figure 4B, 442 peptides scored > 0.5, 332 peptides scored > 0.6, 206 peptides scored > 0.7, 43 peptides scored > 0.8, and 14 peptides scored > 0.9. The closer the peptide ranker score was to 1, the higher the bioactivity of the peptide [47]. In addition, 14 peptides with a score greater than 0.9 were highly abundant in APSH-iii* or APSH-iv*.

The top 14 peptides with the highest PeptideRanker scores were selected for AutoDock Vina molecular docking with ACE and Keap1 proteins to screen for peptides with high biological activity. As shown in Table 5, after molecular docking, all 14 polypeptides could stably exist within the active pocket of the ACE enzyme, with binding energies of −5.8~−9.7 kcal/mol. In addition, all 14 polypeptides could stably exist within the active region of the Keap1 protein, with binding energies of −6.2~−7.8 kcal/mol. According to the reported binding energies between the ACEi peptide, antioxidant peptide, and ligand [25], these binding energy values indicated exceptionally stable ligand–protein interactions. The 14 peptides were input into the common bioactive peptide databases BIOPEP-UWM [48] and EROP-Moscow [49] for matching; all sequences were not successfully matched and were considered new peptide sequences.

### 3.6. Peptide Synthesis and Bioactivity Verification by Wet Experiment

To confirm the biological activities of these 14 peptides, they were synthesized and analyzed. The IC_50_ values of the ACEi activity were in the range of 0.05~0.54 mg/mL, and the ACEi activities of peptide FDRLF (0.05 mg/mL) and peptide FDFRQF (0.12 mg/mL) were the highest (Table 5). Among them, nine peptides had ABTS antioxidant activity, with EC_50_ values of 0.12~1.08 mg/mL, where peptide WGDHGW (0.14 mg/mL) and peptide PDRPW (0.12 mg/mL) had the best ABTS antioxidant activities. Compared with the reported ACEi and antioxidant peptides mentioned in a review by Mao et al. [40], the activities of these peptides were higher.

**Figure 5:**
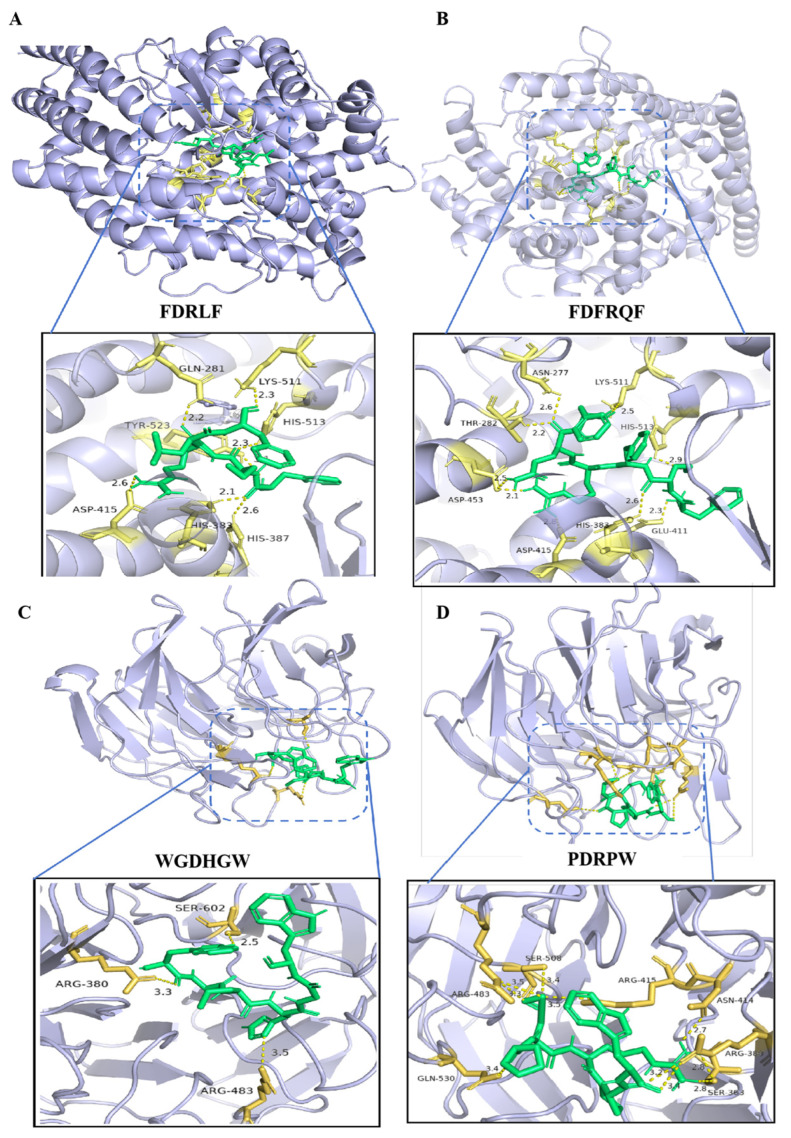
Molecular docking results. (**A**) FDRLF within the active pocket of the ACE enzyme. (**B**) FDFRQF within the active pocket of the ACE enzyme. (**C**) WGDHGW within the active region of the Keap1 protein. (**D**) PDRPW within the active region of the Keap1 protein.

Molecular docking analysis indicated that peptides FDRLF and FDFRQF formed seven and nine hydrogen bonds with the active pocket of ACE, respectively, and peptides WGDHGW and PDRPW formed three and ten hydrogen bonds with the active region of Keap1, respectively (Figure 5). These small molecular peptides rich in hydrophobic and aromatic amino acids can not only enter the active region of enzymes or proteins with small volumes but also form many hydrogen bonds with amino acids in the active region to exert biological activity [50].

## 4. Conclusions

In this study, abalone meat and viscera were used as the raw materials for primary enzymatic hydrolysis. An orthogonal experiment was conducted to improve the utilization rate of abalone proteins and polypeptide yield, resulting in AHIP. Three specific proteases (PEP, α-chymotrypsin, and protease K) were selected for the second hydrolysis on AHIP, producing APSH. A total of 1937 polypeptide sequences were obtained from the highly bioactive components and were virtually screened and analyzed using bioinformatics tools and molecular docking. Finally, 14 ACE inhibitory peptides were obtained, with IC_50_ values of 0.05~0.54 mg/mL, nine of which had good antioxidant activities, with EC_50_ values of 0.12~1.08 mg/mL. In summary, these studies indicated that the combination of enzymatic hydrolysis with specific proteases and virtual screening is an efficient method for mining bioactive peptides. In addition, this study has certain limitations due to the lack of cellular-level investigations. Human *in vivo* models and animal studies could be incorporated for comprehensive analysis in future research.

## Figures and Tables

**Table 1:** Orthogonal table.

Levels	Factors			
	Hydrolysis Time/(h)	Hydrolysis Temperature /(°C)	Solid–Liquid Ratio	Enzyme Dosage /(%)
1	3	37	1:10	1
2	4	46	1:20	2
3	5	55	1:30	3

**Table 2:** Results of orthogonal test.

Experimental Serial Number	Factors				DH/%	Polypeptide Content/%	Comprehensive Score /(Points)
	Hydrolysis Time/(h)	Hydrolysis Temperature/(°C)	Solid–Liquid Ratio	Enzyme Dosage /(%)			
1	1	1	1	1	11.05	78.92	66.15
2	2	2	2	1	15.82	81.59	75.81
3	3	3	3	1	20.35	83.07	84.34
4	3	2	1	2	20.78	70.93	77.76
5	1	3	2	2	19.94	76.52	79.71
6	2	1	3	2	16.66	82.01	77.48
7	2	3	1	3	29.63	63.40	88.16
8	3	1	2	3	19.99	75.52	79.19
9	1	2	3	3	21.58	78.54	83.69
K1	76.517	74.273	77.357	75.433			
K2	80.483	79.087	78.237	78.317			
K3	80.430	84.070	81.837	83.880			
R	3.966	9.797	4.48	8.447			

**Table 3:** Orthogonal validation test.

Validation Experiment Number	DH/%	Polypeptide Content/%
1	21.13	70.72
2	21.61	73.44
3	22.53	73.19
RSD/%	3.27	2.08

**Table 4:** Difference in amino acid composition between A-I and A-V.

AA Species	AA Content/[g·(100 g)^−1^]	
	A-I (2–3 kDa)	A-V (0–1 kDa)
Asp	18.64	13.21
Glu	17.10	9.49
Ser	10.44	5.88
His	4.87	5.00
Gly	17.98	5.03
Thr	8.60	4.82
Arg	4.64	2.00
Ala	7.29	1.82
Tyr	4.20	19.94
Cys-s	1.76	13.11
Val	5.76	2.49
Met	3.51	0.79
Phe	2.90	23.30
Ile	3.03	1.94
Leu	5.94	3.05
Lys	6.45	1.01
Pro	13.67	3.23
Trp	3.83	0.58

**Table 5:** Summary of properties of 14 peptides.

Peptide	Mass/ (Da)	PeptideRanker	Binding Energy with ACE Enzyme /(kcal·mol^−1^)	Binding Energy with Keap1 Protein /(kcal·mol^−1^)	ACEi IC_50_/ (mg·mL^−1^)	ABTS^+^ EC_50_/ (mg·mL^−1^)
FDRLF	696	0.964558	−8.5	−6.8	0.05	-
SPPFFDGMTR	1154	0.951409	−8.1	−6.7	0.18	-
SYPPLGRF	935	0.947818	−8.6	−6.2	0.20	0.17
FDFRQF	858	0.945513	−7.6	−6.4	0.12	-
GFDFRQF	915	0.942461	−6.2	−6.5	0.29	-
WGDHGW	756	0.937728	−7.2	−7.0	0.54	0.14
PEHFPF	772	0.937014	−9.7	−7.8	0.40	-
SSYPPLGRF	1023	0.932763	−8.6	−6.8	0.15	0.16
WPNRPP	765	0.9292	−5.8	−6.8	0.29	0.18
WSDRIPF	919	0.921708	−7.3	−6.8	0.17	0.23
GWDKFWPE	1063	0.920339	−7.3	−7.1	0.33	0.42
PDRPW	669	0.917602	−7.6	−7.6	0.33	0.12
FYDHIF	840	0.917509	−8.9	−7.1	0.37	1.08
ADWDFLPAK	1062	0.910647	−8.2	−7.6	0.23	0.34
SP [18]	202	-	-	-	0.06	-
VDRYF [18]	699	-	-	-	0.28	-

## Data Availability

The original contributions presented in the study are included in the article/Supplementary Material, further inquiries can be directed to the corresponding author.

## Supplementary Materials

The following supporting information can be downloaded at https://www.mdpi.com/article/10.3390/foods14071209/s1, Figure S1: Effect of different hydrolysis conditions on DH of abalone; Figure S2: Hydrolysis effect of abalone powder by specific protease.
