# Supplementary material for: Step Enzymatic Hydrolysis and In Silico Screening-Assisted Preparation of Bioactive Peptides from Abalone
Source: Foods. 2025 Mar 29;14(7):1209. doi: 10.3390/foods14071209 (PMC11988861; doi:10.3390/foods14071209)
Supplement: Supplementary file 1 [file foods-14-01209-s001.zip › foods-3535927-supplementary.pdf]

Supplementary Material

Step enzymatic hydrolysis and *in silico* screening-assisted preparation of bioactive peptides from abalone

Kanzhen Liu [1,2] , Cuiping Pang [2,5] , Qinghua Li [1,2] , Jianghua Li [1,2,3,4] , Guocheng Du [1,2,3,4] and Guoqiang Zhang [1,2,3,4,*]

1. Science Center for Future Foods, Jiangnan University, 1800 Lihu Road, Wuxi, Jiangsu 214122, China; futurefoods@jiangnan.edu.cn
2. School of Biotechnology, Jiangnan University, 1800 Lihu Road, Wuxi, Jiangsu 214122, China; biotech@jiangnan.edu.cn
3. Key Laboratory of Industrial Biotechnology of Ministry of Education, Jiangnan University, 1800 Lihu Road, Wuxi, Jiangsu 214122, China; biotech@jiangnan.edu.cn
4. Jiangsu Province Engineering Research Center of Food Synthetic Biotechnology, Jiangnan University, 1800 Lihu Road, Wuxi, Jiangsu 214122, China; lsyfund@jiangnan.edu.cn
5. Shenzhen Institute of Synthetic Biology, Shenzhen Institute of Advanced Technology, Chinese Academy of Sciences, Shenzhen 518055, China; hcs.edu@siat.ac.cn

* Correspondence: gqzhang@jiangnan.edu.cn (Zhang GQ); Phone: +86-510-85914371, Fax: +86-510-85914371.

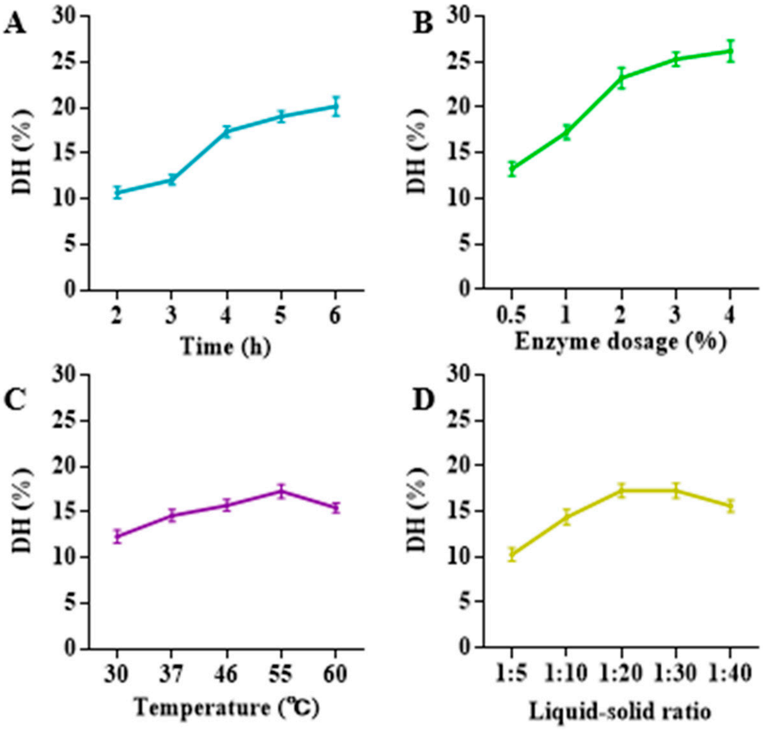

Figure S1. Effect of different hydrolysis conditions on DH of abalone. (**A**) Hydrolysis time. (**B**) Enzyme dosage. (**C**) Hydrolysis temperature. (**D**) Solid-liquid ratio.

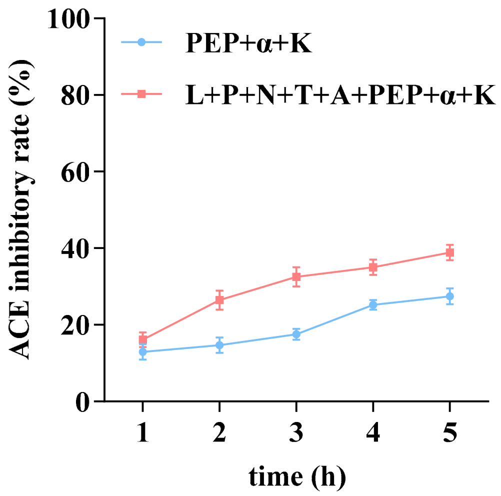

Figure S2. Hydrolysis effect of abalone powder by specific protease. Pep+*α*+K line showed the ACEi activity of the supernatant at 1 mg/mL after the abalone powder was hydrolyzed by 1% (g/g) of the specific proteases.

L+P+N+T+A+Pep+α+K line showed the ACEi activity of the supernatant at 1 mg/mL after abalone powder was hydrolyzed by a mixture of 1% (g/g) of specific proteases and 3% (g/g) of lipase, papain, neutral protease, trypsin and alkaline protease. L: lipase; P: papain; N: neutral protease; T: trypsin; A: alkaline protease; PEP: prolyl endopeptidase; α: α-chymotrypsin; K: proteinase K.
